# Intercellular Adhesion Molecule-1 Enhances Myonuclear Transcription during Injury-Induced Muscle Regeneration

**DOI:** 10.3390/ijms23137028

**Published:** 2022-06-24

**Authors:** Kole H. Buckley, Andrea L. Nestor-Kalinoski, Francis X. Pizza

**Affiliations:** 1 School of Exercise and Rehabilitation Sciences, University of Toledo, 2801 W. Bancroft St., Toledo, OH 43606, USA; kole.buckley@utoledo.edu; 2 Advanced Microscopy & Imaging Center, University of Toledo, Toledo, OH 43606, USA; ander.kalinoski@utoledo.edu

**Keywords:** muscle inflammation, ICAM-1, inflammatory response, regenerating myofibers, myogenic cell fusion

## Abstract

The local inflammatory environment of injured skeletal muscle contributes to the resolution of the injury by promoting the proliferation of muscle precursor cells during the initial stage of muscle regeneration. However, little is known about the extent to which the inflammatory response influences the later stages of regeneration when newly formed (regenerating myofibers) are accumulating myonuclei and undergoing hypertrophy. Our prior work indicated that the inflammatory molecule ICAM-1 facilitates regenerating myofiber hypertrophy through a process involving myonuclear positioning and/or transcription. The present study tested the hypothesis that ICAM-1 enhances global transcription within regenerating myofibers by augmenting the transcriptional activity of myonuclei positioned in linear arrays (nuclear chains). We found that transcription in regenerating myofibers was ~2-fold higher in wild type compared with ICAM-1-/- mice at 14 and 28 days post-injury. This occurred because the transcriptional activity of individual myonuclei in nuclei chains, nuclear clusters, and a peripheral location were ~2-fold higher in wild type compared with ICAM-1-/- mice during regeneration. ICAM-1’s enhancement of transcription in nuclear chains appears to be an important driver of myofiber hypertrophy as it was statistically associated with an increase in myofiber size during regeneration. Taken together, our findings indicate that ICAM-1 facilitates myofiber hypertrophy after injury by enhancing myonuclear transcription.

## 1. Introduction

Restoration of structure and function to skeletal muscle injured by physical activity, trauma, or disease requires completion of a process called muscle regeneration. Muscle regeneration commences with the proliferation of muscle stem cells called satellite cells and culminates in de novo myofiber formation [1,2,3]. During regeneration, nascent (regenerating) myofibers accumulate hundreds of nuclei (myonuclei) that, strikingly, are found in distinctly different cytoplasmic positions [4,5,6]. The majority of myonuclei are situated in centralized linear arrays (nuclear chains) [4,5,7], which defines regenerating myofibers in transverse planes of skeletal muscle. Myonuclei are also found in a peripheral location near the sarcolemma (peripheral myonuclei) or clustered together (nuclear clusters) in regenerating myofibers [4,5,6,7,8]. The extent to which myonuclear accretion and positioning influence the maturation of regenerating myofibers is poorly understood [7].

Myonuclear accretion and positioning during regeneration is accompanied by high rates of protein synthesis during an early stage of regeneration, progressive increases in myofiber size, and ultimately, a restoration of muscle function [5]. In theory, myonuclear accretion promotes hypertrophy of regenerating myofibers by increasing their capacity for transcription. This premise is supported by positive correlations between myonuclear number and regenerating myofiber size [5,7], as well as elevated levels of transcription in regenerating myofibers after injury [7]. A role of myonuclear positioning in the maturation of regenerating myofibers is indicated by the finding that the transcriptional activity of myonuclei in nuclear chains is notably higher than that of peripheral and clustered myonuclei [7]. Furthermore, the number of myonuclei in nuclear chains and the transcription occurring in nuclear chains are strong predictors of an increase in regenerating myofiber size after injury [7]. Thus, the positioning and transcriptional activity of myonuclei in nuclear chains appear to be important in promoting myofiber hypertrophy during regeneration. 

Our research has begun to establish a role of intercellular adhesion molecule-1 (ICAM-1; CD54) in regulation of regenerative and hypertrophic processes within skeletal muscle [5,9,10,11]. ICAM-1 is a transmembrane protein that mediates cell-to-cell adhesion and intra- and inter-cellular communication [10,12,13,14]. ICAM-1 is normally expressed by endothelial cells and leukocytes and other cell types express ICAM-1 during an inflammatory response. Indeed, the inflammatory response in regenerating, hypertrophying, and diseased skeletal muscle is accompanied by myofiber expression of ICAM-1 [5,11,15,16,17,18]. During injury-induced muscle regeneration, ICAM-1 contributes to the regulation of myonuclear number and positioning, as well as facilitates increases in protein synthesis and myofiber size [5]. Importantly, the absence of ICAM-1 during regeneration results in aberrant myonuclear positioning and uncoupled myonuclear number from indices of myofiber hypertrophy [5]. Our findings indicated that ICAM-1 facilitates regenerating myofiber hypertrophy through a process involving myonuclear positioning and transcription. 

Our initial objective was to substantiate and extend our findings on the influence of ICAM-1 on myonuclear accretion and positioning during injury-induced muscle regeneration [5]. Our primary objective, however, was to test the hypothesis that ICAM-1 enhances global transcription within regenerating myofibers by augmenting the transcriptional activity of myonuclei situated within nuclear chains. The goal of this objective was to provide insight into how ICAM-1 facilitates hypertrophy of regenerating myofibers. Our objectives were achieved by quantifying myonuclear number, positioning, and transcriptional activity in single myofibers of wild type mice and in mice with a germline mutation for ICAM-1 (ICAM-1-/-) [5,11]. 

## 2. Methods

### 2.1. Mice

Adult (12–16 weeks of age) male and female wild type (C57BL/6) and ICAM-1-/- (Icam1tm1^cws^) [5,11] mice were used in this study. The ICAM-1-/- mice have on occasion been backcrossed with C57BL/6. We previously demonstrated that skeletal muscle of ICAM-1-/- mice develop normally [5,11]. That is, body mass, muscle mass, muscle protein content, myofiber size, and muscle function are similar between adult wild type and ICAM-1-/- mice under control conditions [5,11].

Mice were housed and bred in an accredited animal facility at the University of Toledo. Mice were fed standard laboratory chow and water ad libitum and were exposed to a 12-h light-dark cycle. Isoflurane was used as an anesthetic for surgical procedures and mice were sacrificed via cervical dislocation. All procedures were approved by the institutional animal care and use committee.

### 2.2. Muscle Injury

We exposed gastrocnemius muscles through a small skin incision and then intramuscularly injected a total of 50 μL of 1.2% barium chloride (Sigma-Aldrich), with each head of the gastrocnemius receiving 25 μL [7]. Skin incisions were sutured closed, and mice resumed normal cage activity. Gastrocnemius muscles were collected from euthanized mice that did not experience muscle injury (0 days post-injury) and from mice at 7, 14, and 28 days post-injury.

### 2.3. Myofiber Isolation and Fixation

We used published procedures for isolating single myofibers from gastrocnemius muscles [19,20]. Single myofibers were liberated from gastrocnemius muscles by enzymatically digesting them in 0.18% collagenase type 1 (Sigma-Aldrich; St. Louis, USA). Isolated myofibers were fixed by transferring them to a tube containing 4% formaldehyde in phosphate-buffered saline (PBS) for 30 min and then rinsed with PBS. Individual myofibers that did not show signs of hypercontraction were randomly selected and carefully placed on slides coated with a solution containing 0.04% chromium (III) potassium sulfate and 0.4% gelatin. 

### 2.4. Global Transcription in Myonuclei 

Transcription was assessed through the use of 5-ethynyluridine (EU), a uridine analog that can be incorporated into nascent RNA during transcription [21]. The incorporation of EU into RNA is a global measure of transcription, as it is not specific to a type of RNA (e.g., rRNA and mRNA). Mice received 2 mg of EU in sterile PBS via i.p. injection 5 h before muscle collection [7,22]. 

We used reagents and procedures in the Click-iT^TM^ RNA Imaging Kit (Invitrogen) to detect EU within myofibers. As suggested by the manufacturer, background detection of EU was revealed by omitting the catalyst for the copper mediated reaction from the procedures. Myonuclei were stained with DRAQ-5 (1:1000; Invitrogen) and slides were mounted in Fluoromount G (SouthernBiotech; Birmingham, AL, USA).

### 2.5. Image Acquisition

Myofibers were imaged via confocal microscopy (Leica TCS SP5 multiphoton confocal microscope). Non-regenerating myofibers from control (0 days post-injury) muscles and regenerating myofibers from injured muscles were imaged. The same settings (e.g., exposure time) were used to capture all images. The entire depth of a myofiber was imaged in 2 μm increments. A maximum z-projection was produced by merging all images in a single z-stack. 

### 2.6. Quantification of Myonuclear Number, Position, and Transcriptional Activity

Quantification of dependent measures in maximum z-projections were performed as previously described [7]. Briefly, an outline was created of individual myonuclei using Image Pro 7 software (Media Cybernetics). The total number of myonuclei, as well as the number of myonuclei in chains, clusters, and a peripheral location were counted. Myonuclei situated in the same x and y position, but a different z-plane would have been indistinguishable and therefore counted as a single myonuclei in our analysis of z-projections. A nuclear chain was defined as a series of five or more myonuclei that were organized in a linear array near the center of myofibers. A nuclear cluster was defined as a non-linear grouping of three or more myonuclei. Peripheral myonuclei were defined as a myonucleus not localized to a chain or cluster. Myonuclei at the ends of myofibers and nuclei in contact with myofibers or outside their boundary were excluded from the analysis. This was done to exclude nuclear clusters near the myotendinous junction, as well as to exclude cells that were closely associated with the membrane of myofibers (e.g., myoblasts, leukocytes, and satellite cells) from our analysis. Myonuclei that normally cluster in the neuromuscular junction [23,24] were included in our analysis, as we analyzed the middle region of myofibers. As previously noted, nuclear clusters in regenerating myofibers are not restricted to the neuromuscular junction [7].

The mean fluorescent intensity (MFI) of EU within outlines of individual myonuclei, as well as the area (µm^2^) of outlines were quantified using Image Pro 7. The corrected integrated density of EU for individual myonuclei was calculated using the following equation: (Myonuclear area×MFI)−(Myonuclear area×MFI−background). This value (integrated density/myonucleus) was used to represent the transcriptional activity of individual myonuclei.

The portion of a myofiber that was used in our quantitative analysis was measured for length and average width using cellSens software (Olympus Life Sciences). Myofiber volume was calculated using the following equation: ($Myofiber\;volume=\pi \times average\;radius^{2}\times length\;of\;myofiber\;segment)$. 

At least 4 myofibers from 3 different muscles per time point were analyzed (*n* = 14–19 myofibers/time point). On average, 3243 μm of myofiber length (SD = ±889) and 579 (SD = ±248) myonuclei per myofiber were analyzed (*n* = 124 myofibers). The total number of myonuclei analyzed was 71,841.

### 2.7. Statistics

Data sets from wild type and ICAM-1-/- mice were analyzed using a two-way ANOVA (Sigma Plot; Systat). Grouping factors for the 2-way ANOVA were genotype (wild type and ICAM-1-/-) and post-injury time point. The Newman-Keuls post-hoc test was then used to locate differences between groups when the observed F ratio was statistically significant (*p* < 0.05). Bivariate and multiple regression analysis was performed using Sigma Plot to examine relationships between dependent measures. Dependent measures included in forward stepwise multiple regression analysis included the number of myonuclei or integrated density for each myonucleus in nuclear chains, peripheral, and clustered positions with a *p* < 0.05 required to enter the model. Data are reported as Mean ± SEM.

## 3. Results

Myonuclear number, positioning, and transcriptional activity in wild type mice were reported in our recent publication [7]. These data, which were collected at the same time measurements were made in myofibers of ICAM-1-/- mice, are included in the present report. This was done so that the contribution of ICAM-1 to myonuclear accretion, positioning, and transcriptional activity during regeneration could be determined. Below we present only comparisons between the genotypes, as the temporal response in wild type mice was presented and discussed in our recent publication [7]. 

### 3.1. ICAM-1 Alters Myonuclear Number and Density during Regeneration

**Figure 1:**
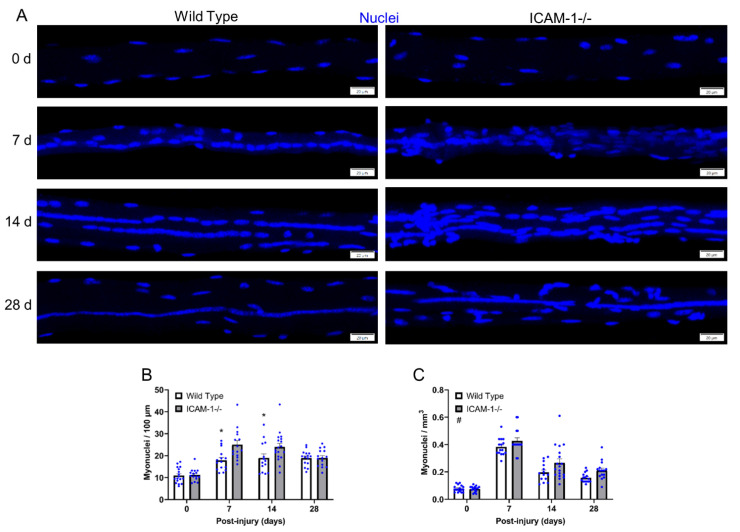
Myonuclear number and density before and during injury-induced muscle regeneration. (**A**) z-projection images of myonuclei (blue) in myofibers isolated at 0, 7, 14, and 28 days post-injury. Scale bars = 20 μm. (**B**) The number of myonuclei expressed relative to myofiber length (100 μm). (**C**) The number of myonuclei expressed relative to myofiber volume (mm^3^). *n* = 14–18 myofibers/time point for each genotype. * = lower for Wild Type compared with ICAM-1-/- mice at indicated time point (interaction effect; *p* = 0.018). # = lower for Wild Type compared with ICAM-1-/- mice (main effect for genotype; *p* = 0.002).

We first sought to substantiate and extend our initial findings on the extent to which ICAM-1 influences myonuclear number during regeneration [5]. This was done by quantifying myonuclear number in single myofibers of wild type and ICAM-1-/- mice (Figure 1A) and expressing it relative to myofiber length (Figure 1B) or volume (Figure 1C). 

ICAM-1 did not influence the number (myonuclei/100μm) or density (myonuclei/mm^3^) of myonuclei in control myofibers (0 days post-injury), which is consistent with our prior work [5]. During regeneration, myonuclear number (myonuclei/100 μm) was 21%–29% lower in wild type compared with ICAM-1-/- mice at 7 and 14 days post-injury (interaction *p* = 0.018). Myonuclear density (myonuclei/mm^3^) for wild type mice was also lower than levels observed in ICAM-1-/- mice throughout regeneration (main effect *p* = 0.002). Differences between the genotypes in myonuclear density reflect the finding that myofibers of ICAM-1-/- mice had more myonuclei (myonuclei/100 μm) and were smaller during regeneration [5].

Our findings confirmed that the absence of ICAM-1 during regeneration elevates myonuclear number in regenerating myofibers [5]. This indicates that either ICAM-1 inhibits myonuclear accretion during regeneration, or that the absence of ICAM-1 resulted in a compensatory increase in myonuclear accretion. 

### 3.2. ICAM-1 in Myonuclear Positioning during Regeneration

**Figure 2:**
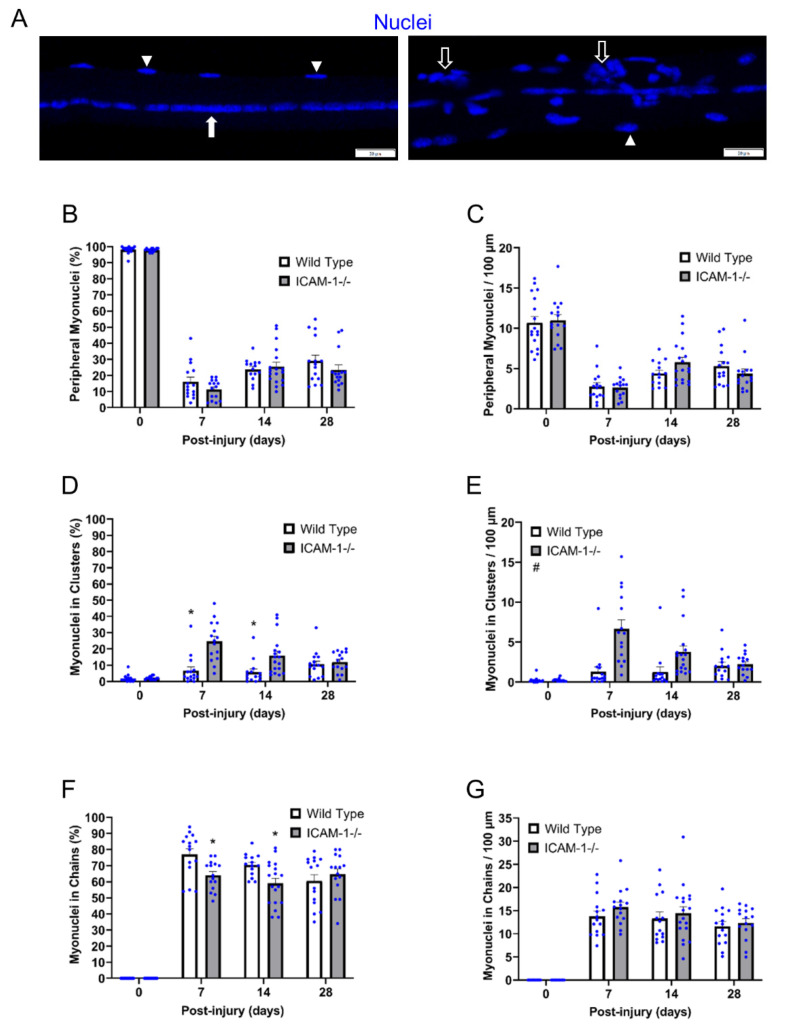
Myonuclear positioning before and during injury-induced muscle regeneration. (**A**) z-projection images of myonuclei (blue) positioned in chains (filled arrow), clusters (open arrow), and a peripheral position (arrowhead) during regeneration. Scale bar = 20 µm. (**B**) The number of peripheral myonuclei expressed as a percentage of the total number of myonuclei within a myofiber and (**C**) relative to 100 μm of myofiber length. (**D**) The number of myonuclei in chains expressed as a percentage of the total number of myonuclei within a myofiber and (**E**) relative to 100 μm of myofiber length. * = lower for Wild Type compared with ICAM-1-/- mice at indicated time point (interaction effect; *p* = 0.014. (**F**) The number of myonuclei in clusters expressed as a percentage of the total number of myonuclei within a myofiber and (**G**) relative to 100 μm of myofiber length. * = lower for wild type compared with ICAM-1-/- mice at indicated time point (interaction effect; *p* ≤ 0.001. # = lower for Wild Type compared with ICAM-1-/- mice (main effect; *p* ≤ 0.001. *n* = 14–18 myofibers/time point for each genotype.

We quantified the number of myonuclei in nuclear chains and clusters, as well as in a peripheral position to characterize the extent to which ICAM-1 influences myonuclear positioning during regeneration (Figure 2A). This was done by expressing the number of myonuclei in each myonuclear position relative to the total number of myonuclei within a myofiber and relative to myofiber length. 

The normal peripheral positioning of myonuclei in control myofibers was similar between the genotypes (Figure 2B,C). Very few-clustered myonuclei (<2%) (Figure 2D,E) and no nuclear chains (Figure 2F,G) were observed in control myofibers of wild type and ICAM-1-/- mice. These findings demonstrate ICAM-1 does not influence myonuclear positioning in non-regenerating myofibers of control mice. 

Like wild type mice, the majority of myonuclei in regenerating myofibers of ICAM-1-/- mice resided in nuclear chains. However, the percentage of myonuclei in nuclear chains was ~20% higher for wild type compared with ICAM-1-/- mice at 7 and 14 days post-injury (interaction *p* = 0.014; Figure 2F). On the other hand, the number of myonuclei in nuclear chains expressed relative to myofiber length was similar between the genotypes during regeneration (Figure 2G).

Compared with nuclear chains, fewer peripheral and clustered myonuclei were found in regenerating myofibers. Both the percentage and number of peripheral myonuclei during regeneration were similar between the genotypes during regeneration (Figure 2B,C). In contrast, the percentage and number of myonuclei in nuclear clusters was on average 2.3–2.7 fold lower for wild type compared with ICAM-1-/- mice after injury (main effect *p* < 0.001; Figure 2D,E). The aberrant myonuclear clustering in ICAM-1-/- mice was not limited to the middle region of myofibers where the neuromuscular junction typically resides. Importantly, the increased number of clustered myonuclei in regenerating myofibers of ICAM-1-/- mice explained 74% and 49% of the absolute difference in myonuclei number (myonuclei/100 μm; Figure 1B) between the genotypes at 7 and 14 days post-injury, respectively. This indicates that differences in myonuclear number between the genotypes during regeneration were primarily attributable a greater number of clustered myonuclei in ICAM-1-/- mice.

We conclude that ICAM-1 does not influence the positioning of myonuclei in nuclear chains or in a peripheral location during regeneration. Rather, ICAM-1 appears to have a role in minimizing nuclear clustering during regeneration. This interpretation is based on the finding that the absence of ICAM-1 increased myonuclear clustering in regenerating myofibers, which in turn elevated the number of myonuclei in regenerating myofibers.

### 3.3. The Loss of ICAM-1 Impairs the Relationship between Myonuclear Number and Myofiber Size

Through correlational analysis of myonuclear number and myofiber area, we reported that ICAM-1 enhanced the responsiveness of regenerating myofibers to myonuclei [5]. We extend our initial findings by determining the extent to which ICAM-1 influences the relationship between myonuclear number and myofiber volume, as well as the relationship between myonuclear positioning and myofiber volume. 

**Figure 3:**
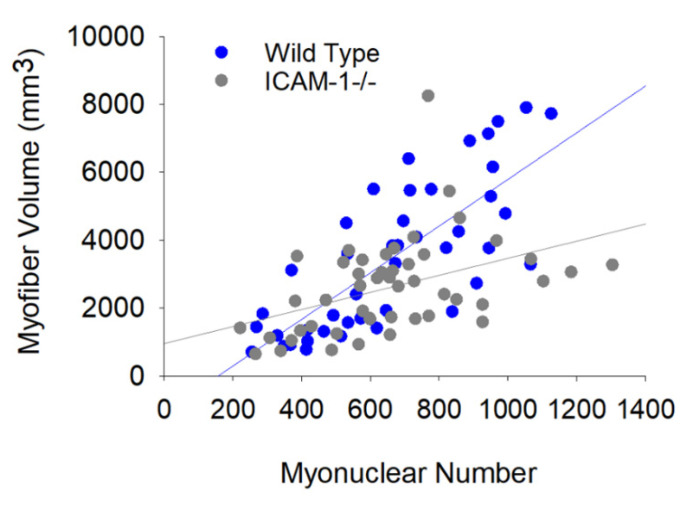
The relationship between myonuclear number and myofiber volume in Wild Type and ICAM-1-/- mice during injury-induced muscle regeneration. The scatter plot and regression lines display the number of myonuclei and corresponding myofiber volume (mm^3^) within a segment for 7–28 days post-injury. Data and regression line for Wild Type mice (r = 0.77; Myofiber volume = 0.66 × number of myonuclei −88.0; *n* = 44 myofibers) and ICAM-1-/- mice (r = 0.43; Myofiber volume = 0.25 × number of nuclei + 99.2; *n* = 48 myofibers).

Bivariate regression analysis revealed that the relationship between myonuclear number and myofiber volume for wild type mice (r = 0.77; *p* < 0.001) was stronger than that observed in ICAM-1-/- mice (r = 0.43; *p* = 0.003) during regeneration (Figure 3). This was also apparent when analyzing data at each post-injury time point (Table S1). The slope of the regression line was also higher in wild type compared with ICAM-1-/- mice, particularly at 14 days post-injury. This finding is in agreement with our previously published results [5].

Stepwise multiple regression analysis of wild type mice revealed that the number of myonuclei in nuclear chains was a strong predictor of myofiber volume during regeneration (Table S2). In wild type mice, myonuclei in nuclear chains was the only myonuclear position that predicted myofiber volume at 7 and 14 days post-injury, whereas the inclusion of peripheral myonuclei strengthened the prediction of myofiber volume at 28 days post-injury (Table S2). Importantly, the number of myonuclei in nuclear chains of ICAM-1-/- mice failed to predict myofiber volume at 7 and 14 days post-injury. At 28 days post-injury, the inclusion of myonuclei in nuclear chains of ICAM-1-/- mice strengthened the predictive value of peripheral myonuclei.

Our findings demonstrated that ICAM-1 strengthens the association between myonuclear number and regenerating myofiber size, and to some extent the responsiveness of regenerating myofibers to myonuclei. As the absence of ICAM-1 negated the contribution of nuclear chains to the prediction of myofiber volume, ICAM-1’s role in facilitating regenerating myofiber hypertrophy appears to involve the function of myonuclei in nuclear chains. 

### 3.4. The Loss of ICAM-1 Reduces Transcriptional Activity of Myonuclei in Regenerating Myofibers

**Figure 4:**
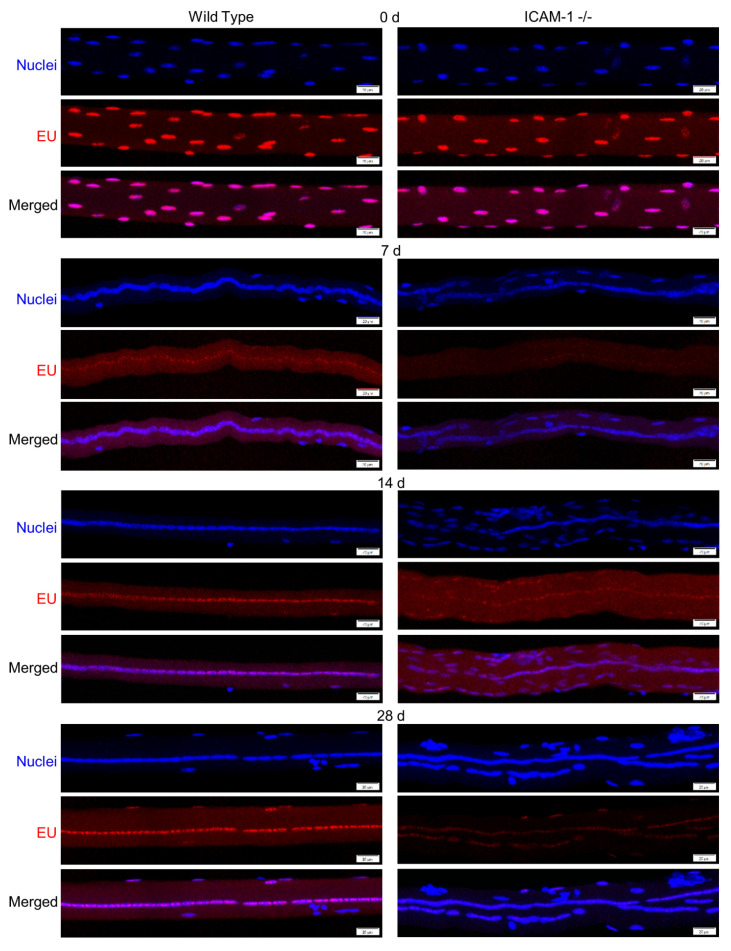
Detection of nascent RNA in myofibers before and during injury-induced muscle regeneration. Mice were administered 5-ethynyluridine (EU; red) and its presence detected within myonuclei (blue) of myofibers. Images are representative of responses observed 0, 7, 14, and 28 d post-injury. Scale bars = 20 μm.

We previously demonstrated that ICAM-1 promotes an increase in protein synthesis and myofiber size during regeneration [5]. To gain insight into the involvement of transcription in ICAM-1-mediated protein synthesis [5], we quantified the incorporation of EU into nascent RNA of individual myonuclei (Figure 4). This provided a global measure of myonuclear transcription and did not provide insight into the type of RNA (e.g., rRNA and mRNA) being transcribed [21,22].

**Figure 5:**
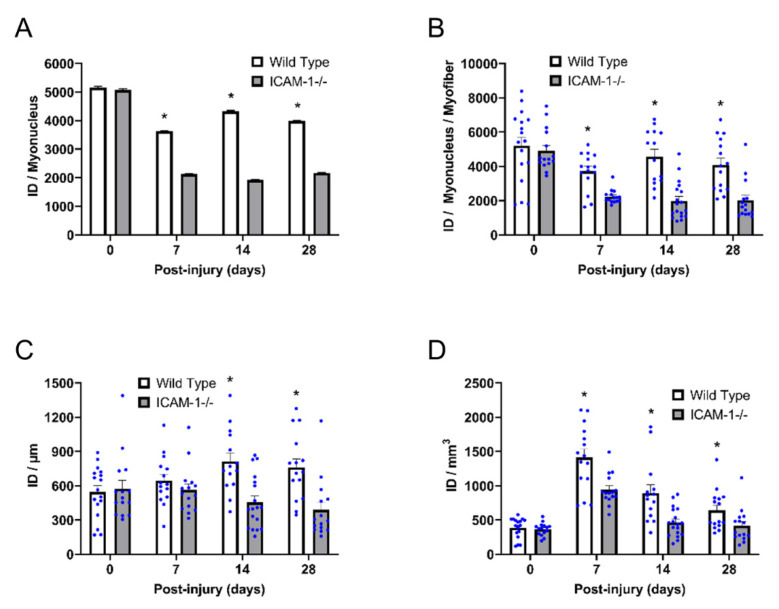
Transcriptional activity of individual myonuclei and myofibers before and during injury-induced muscle regeneration. An integrated density (ID) of EU was calculated for each myonucleus to represent its transcriptional activity, as described in the Methods. (**A**) Integrated density for all myonuclei within myofibers at 0, 7, 14, and 28 days post-injury (*n* = 5310–13,057 myonuclei per time point/genotype). * = higher for Wild Type compared with ICAM-1-/- mice at indicated time point (interaction effect; *p* ≤ 0.001). (**B**) Integrated density for all myonuclei when averaged on a per myofiber basis (*n* = 14–18 myofibers per time point/genotype. * = higher for Wild Type compared to ICAM-1-/- mice at indicated time point (interaction effect; *p* ≤ 0.001). (**C**) Integrated density per myofiber length (100 μm) (*n* = 14–18 myofibers per time point/genotype). * = higher for Wild Type compared with ICAM-1-/- mice at indicated time point (interaction effect; *p* = 0.003). (**D**) Integrated density per myofiber volume (mm^3^) (*n* = 14–18 myofibers per time point/genotype). * = higher for Wild Type compared with ICAM-1-/- mice at indicated time point (interaction effect; *p* = 0.009).

Transcriptional activity of myonuclei (integrated density/myonucleus) within control myofibers was similar between the genotypes (Figure 5A). During regeneration however, integrated density/myonucleus was 2-fold greater for wild type compared with ICAM-1-/- mice at all post-injury time points (interaction *p* < 0.001). Our finding was consistent when an average integrated density of individual myonuclei was calculated within a myofiber (interaction *p* < 0.008; Figure 5B). This finding demonstrates that the loss of ICAM-1 reduces the transcriptional activity of individual myonuclei during regeneration. 

### 3.5. The Loss of ICAM-1 reduces the Collective Transcriptional Activity of Regenerating Myofibers

ICAM-1’s role in stimulating protein synthesis in regenerating myofibers would likely stem from the collective transcriptional activity of myonuclei, as opposed to the transcriptional activity of individual myonuclei. Thus, we calculated the total transcriptional activity within a myofiber by summing the integrated density for all myonuclei within a myofiber. Total integrated density was then expressed relative to myofiber length (integrated density/μm) or volume (integrated density/mm^3^). 

Total transcriptional activity (integrated density/μm) was ~2 fold higher in regenerating myofibers of wild type compared with ICAM-1-/- mice at 14 and 28 days post-injury (interaction *p* = 0.003; Figure 5B). This indicates that the increased number of myonuclei within regenerating myofibers of ICAM-1-/- (Figure 1B) failed to compensate for the reduced transcriptional activity of individual myonuclei (Figure 5A). In contrast to wild type mice, total transcriptional activity in regenerating myofibers of ICAM-1-/- mice remained at control levels throughout the course of regeneration. This finding indicates that ICAM-1 is necessary for increased transcriptional activity in regenerating myofibers. 

Total integrated density expressed relative to myofiber volume (integrated density/mm^3^) was also different between the genotypes after injury (Figure 5C). Specifically, integrated density/mm^3^ was 1.5–2.0 fold higher in wild type compared with ICAM-1-/- mice during regeneration (interaction *p* = 0.009). Differences in integrated density/mm^3^ between the genotypes reflect the greater transcriptional activity occurring in regenerating myofibers of wild type mice (Figure 5B), as myofiber size is also greater in wild type compared with ICAM-1-/- mice during regeneration [5].

Our findings demonstrated that the loss of ICAM-1 reduces the collective transcriptional activity of regenerating myofibers after injury. Interestingly, the greater number of myonuclei within regenerating myofibers of ICAM-1-/- compared with wild type mice failed to restore transcriptional activity to wild type levels. A similar failure was observed for protein synthesis and myofiber size in our prior work [5].

### 3.6. ICAM-1 Does Not Influence the Positional Context of Myonuclear Transcription

Next, we determined the extent to which ICAM-1 influences the positional context of transcription during regeneration. This was achieved in two ways. One, we partitioned the integrated density of individual myonuclei according to their position within a myofiber. Two, we expressed the total integrated density for each myonuclear position within a myofiber relative to myofiber length. 

**Figure 6:**
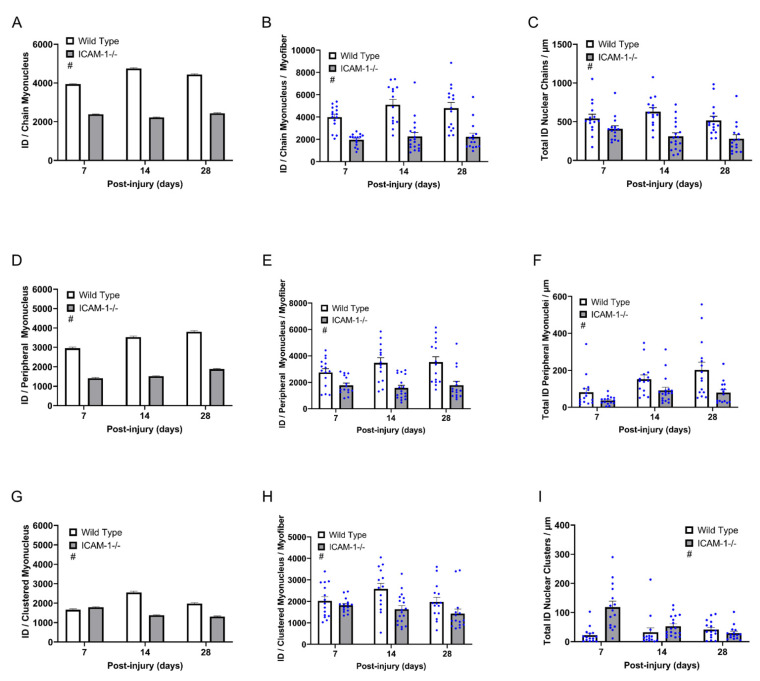
Positional context of transcriptional activity of individual myonuclei before and during injury-induced muscle regeneration. An integrated density (ID) of EU was calculated for each myonucleus to represent its transcriptional activity, as described in the Methods. (**A**) Integrated density per chain myonucleus (*n* = 5529–7850). (**B**) Integrated density per chain myonucleus when averaged on a per myofiber basis (*n* = 14–18 myofibers per time point/genotype. (**C**) Total integrated density of chain myonuclei per myofiber length (μm) (*n* = 14–18 myofibers per time point/genotype). (**D**) Integrated density per peripheral myonucleus (*n* = 891–3230). (**E**) Integrated density per peripheral myonucleus when averaged on a per myofiber basis (*n* = 14–18 myofibers per time point/genotype. (**F**) Total integrated density of peripheral myonuclei per myofiber length (μm) (*n* = 14–18 myofibers per time point/genotype). (**G**) Integrated density per clustered myonucleus (*n* = 594–2386). (**H**) Integrated density per clustered myonucleus when averaged on a per myofiber basis (*n* = 14–18 myofibers per time point/genotype (**I**) Total integrated density of clustered myonuclei per myofiber length (μm) (*n* = 14–18 myofibers per time point/genotype). # = higher for Wild Type compared to ICAM-1-/- mice (main effect; *p* ≤ 0.001) for (**A**–**G**). # = higher for ICAM-1-/- compared to Wild Type (main effect; *p* ≤ 0.001) for (**I**).

The average integrated density of individual myonuclei in nuclear chains was 1.9 fold higher for wild type compared with ICAM-1-/- mice during regeneration (main effect *p* < 0.001; Figure 6A). This was also true when an average integrated density of individual myonuclei in nuclear chains was calculated within a myofiber (main effect *p* < 0.001; Figure 6B). Similar to wild type mice, most of the transcription in regenerating myofibers of ICAM-1-/- mice was occurring in nuclear chains (Figure 6C). However, the total transcription activity of myonuclei in nuclear chains (integrated density nuclear chains/µm) was on average 1.7-fold higher in wild type compared with ICAM-1-/- mice during regeneration (main effect *p* < 0.001). These findings demonstrated that the absence of ICAM-1 reduces the transcriptional activity of myonuclei in nuclear chains during regeneration. 

Similar to nuclear chains, the average integrated density of individual peripheral myonuclei during regeneration was 2.1 fold higher in wild type compared with ICAM-1-/- mice (main effect *p* < 0.001; Figure 6D). Findings were consistent when the average integrated densities of individual peripheral myonuclei per myofiber were calculated (main effect *p* < 0.001; Figure 6E). The total transcriptional activity of peripheral myonuclei (integrated density peripheral myonuclei/µm) was on average 2.1 fold higher in wild type compared with ICAM-1-/- mice during regeneration (main effect *p* < 0.001; Figure 6F). These findings demonstrated that the loss of ICAM-1 reduces the transcriptional activity of peripheral myonuclei during regeneration. 

The average integrated density of individual myonuclei in nuclear clusters during regeneration was low in both genotypes (Figure 6G). The transcriptional activity of myonuclei in nuclear clusters of wild type mice was on average 1.4 fold higher than levels observed in ICAM-1-/- mice (main effect *p* < 0.001). Likewise, the average integrated density of individual myonuclei in clusters per myofiber displayed the same results (Figure 6H). Despite the higher activity of individual myonuclei in nuclear clusters of wild type mice, total transcriptional activity within nuclear clusters (integrated density of nuclear clusters/µm) was on average 2.1 fold lower in wild type compared with ICAM-1-/- mice (main effect *p* < 0.001; Figure 6I). This finding primarily reflects the 2.7 fold lower number of clustered myonuclei (clustered myonuclei/100 µm) in regenerating myofibers of wild type compared with ICAM-1-/- mice during regeneration. Thus, the reduced transcriptional activity of clustered myonuclei in regenerating myofibers of ICAM-1-/- mice was offset by an increase in the number of clustered myonuclei during regeneration. Overall, these findings indicate that the absence of ICAM-1 reduces the transcriptional activity of individual myonuclei in nuclear clusters. 

Our findings demonstrate that ICAM-1 influences myonuclear transcription during regeneration in a manner that is not specific to a myonuclear position. Indeed, the absence of ICAM-1 reduced the transcriptional activity of myonuclei in nuclear chains, clusters, and a peripheral location during regeneration by a similar magnitude. 

### 3.7. The Loss of ICAM-1 Impairs the Relationship between Transcription and Myofiber Size

**Figure 7:**
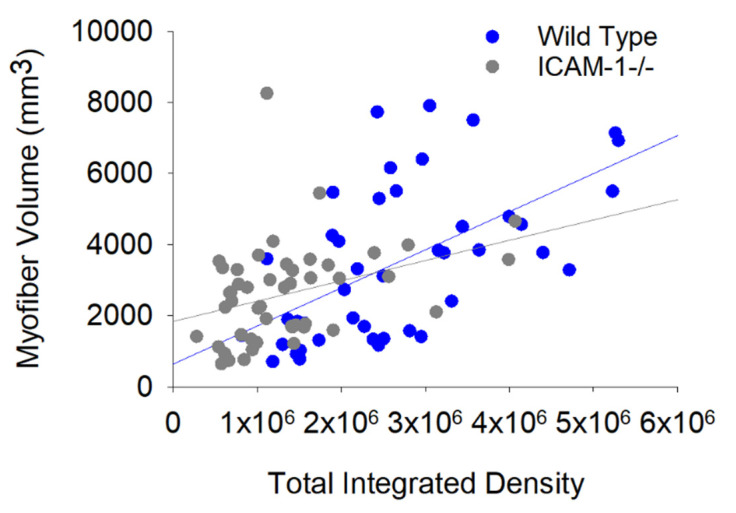
The relationship between integrated density and myofiber volume in Wild Type and ICAM-1-/- mice during injury-induced muscle regeneration. The scatter plot and regression lines display the total integrated density and corresponding myofiber volume (mm^3^) within a segment for 7–28 days post-injury. Data and regression line for Wild Type mice (r = 0.59; Myofiber volume = 0.0011× +634.80; *n* = 44 myofibers) and ICAM-1-/- mice (r = 0.38; Myofiber volume = 0.0006× +1702.76; *n* = 49 myofibers).

We explored the impact of ICAM-1’s enhancement of transcription on myofiber hypertrophy during regeneration by performing correlational analyses. Bivariate regression analysis revealed that the relationship between total integrated density and myofiber volume for wild type mice (r = 0.64; *p* < 0.001; Figure 7) was stronger than that observed in ICAM-1-/- mice (r = 0.39; *p* = 0.06) during the course of regeneration. Stepwise multiple regression analysis demonstrated that the collective transcriptional activity of myonuclei in nuclear chains predicted myofiber volume in wild type, but not ICAM-1-/- mice (Table S3). Only the transcriptional activity of peripheral myonuclei predicted myofiber volume in ICAM-1-/- mice. These findings are consistent with the premise that ICAM-1 mediated transcription, particularly in nuclear chains, facilitates hypertrophy of regenerating myofibers. 

## 4. Discussion

The present study provides a deeper understanding of the immunobiology of muscle regeneration by demonstrating the involvement of ICAM-1 in the regulation of myonuclear transcription during injury-induced muscle regeneration. We report for the first time that ICAM-1 is necessary for increased transcription in regenerating myofibers after injury. This requirement was independent of myonuclear positioning, as ICAM-1 enhanced the transcriptional activity of individual myonuclei in nuclei chains, nuclear clusters, and a peripheral location during regeneration. ICAM-1’s enhancement of transcription, particularly in nuclear chains, was statistically associated with an increase in myofiber size during regeneration. These novel findings, coupled with results from our prior work [5,9,11], indicated that ICAM-1 facilitates protein synthesis and myofiber hypertrophy after injury by enhancing myonuclear transcription.

Previous investigators have established that regenerating myofiber formation after injury is dependent on progenitor cells (e.g., myoblasts) derived from satellites cells [25,26,27] and the expression of myomaker and myomixer (also known as myomerger and minion) by myoblasts [28,29]. Once formed, nascent myotubes/regenerating myofibers add hundreds of myonuclei, with the majority of them positioned in centralized nuclear chains [5]. Importantly, the molecules and pathways that regulate myonuclear accretion and positioning in regenerating myofibers are obscure. Furthermore, the extent to which myonuclear number and positioning influence functional activities of myonuclei and the phenotype of the regenerating myofibers has yet to be established.

A major finding of the present study is that ICAM-1 increased the collective transcriptional activity of regenerating myofibers after injury. This was achieved by ICAM-1 enhancing the transcriptional activity of individual myonuclei in nuclear chains, as well as in individual peripheral and clustered myonuclei. Thus, ICAM-1 did not influence the normal positional context of myonuclear transcription in regenerating myofibers. Because most of the transcription in regenerating myofibers occurs in nuclear chains, ICAM-1’s enhancement of transcription in nuclear chains likely drives phenotypic changes within regenerating myofibers. This speculation is supported by our correlational analyses. Namely, the absence of ICAM-1 negated the contribution of transcription in nuclear chains to increases in regenerating myofiber size after injury. Furthermore, we previously demonstrated that the absence of ICAM-1 impaired increases in protein synthesis and myofiber size during regeneration [5]. Interestingly, both the collective transcriptional activity of regenerating myofibers (present study) and rates of muscle protein synthesis [5] remained at control levels for ICAM-1-/- mice during regeneration. Taken together, our findings indicate that ICAM-1 mediated transcription facilitates regenerating myofiber hypertrophy after injury.

ICAM-1 could enhance myonuclear transcription, as well as downstream processes of muscle regeneration by regulating multiple processes. In cultured cells (e.g., endothelial cells and leukocytes) [13,14], ligation of membrane ICAM-1 activates transcription factors (e.g., AP-1 and FAST-1) [30,31], membrane receptors (e.g., IGF-1R and HGFR) [31], non-receptor kinases (e.g., Src family) [32], signaling molecules (e.g., MAPK, Akt, and Rho GTPases) [33,34,35,36], and cytoskeletal-associated proteins (e.g., focal adhesion kinase and paxillin) [34]. Ligation of membrane ICAM-1 also increases expression of early response genes (e.g., c-fos) [37], cytokines (e.g., IL-1ß and MIP-1α) [30,38,39], and adhesion molecules (e.g., VCAM-1 and ICAM-1) [40,41]. Soluble ICAM-1, which is generated through proteolytic cleavage of membrane ICAM-1 and alternative splicing of the ICAM-1 gene, has also been reported to enhance macrophage chemotaxis and cytokine production [42,43,44]. Which, if any, of the reported actions of ICAM-1 facilitates increases in transcription, protein synthesis, and myofiber size during regeneration remains to be determined. In theory, ICAM-1 signaling in cell types that reside in injured skeletal muscle (e.g., myofibers, endothelial cells, and leukocytes) [5,11] could alter the extracellular milieu of regenerating muscle, which in turn facilitates molecular and cellular processes of regenerating myofiber hypertrophy. As forced expression of ICAM-1 by cultured myotubes augmented Akt/p70s6k signaling and protein synthesis [9], it is also conceivable that ICAM-1 signaling in regenerating myofibers contributes to the regulation of transcription and protein synthesis. Further investigation is needed to determine how ICAM-1 facilitates molecular and cellular processes of myofiber hypertrophy during regeneration. This includes determining the specific contribution of ICAM-1 expressed by myogenic cells, leukocytes (e.g., macrophages), and endothelial cells to injury-induced muscle regeneration.

The present study clarifies and extends our prior findings on the role of ICAM-1 in myonuclear accretion and positioning during regeneration [5]. We initially found that the absence of ICAM-1 during regeneration increased myonuclear number and clustering, while reducing nuclear chain length [5]. The present study revealed that the increased number of myonuclei in regenerating myofibers of ICAM-1-/- mice was attributable to an increased number of clustered myonuclei. Importantly, ICAM-1 did not influence the number of myonuclei in nuclear chains and in a peripheral location during regeneration. These findings indicate that ICAM-1 minimizes nuclear clustering without influencing the position of ~90% of the myonuclei within regenerating myofibers.

The physiological significance of myonuclear clustering and its increased prevalence in ICAM-1-/- mice during regeneration is unclear. Myonuclei in developing myotubes/myofibers normally cluster together during an early stage of in vitro myogenesis [45,46,47], which is characterized by a rapid rate of myogenic cell fusion/myonuclear accretion. Thus, nuclear clusters observed in the present study could represent myonuclei that were recently added to regenerating myofibers. If true, then the increased myonuclear clustering in regenerating myofibers of ICAM-1-/- mice could reflect a compensatory increase in myonuclear accretion. On the other hand, myonuclear clustering during in vitro myogenesis is followed by myonuclear dispersion, which is regulated by the dynamic interplay between components of LINC (Linker of Nucleoskeleton and Cytoskeleton) complex, motor proteins, and the cytoskeleton [45,46,47]. Specifically, inhibition of the LINC complex, motor proteins, or microtubules increases myonuclear clustering and impairs their motility within cultured myotubes/myofibers [48,49,50,51,52,53,54]. Thus, the aberrant myonuclear clustering observed in ICAM-1-/- mice could also represent myonuclei that have yet to or are unable to move to central or peripheral position within regenerating myofibers. If this scenario were true, then the number of myonuclei in nuclear chains and/or in a peripheral position would be expected to be lower in ICAM-1-/- mice during regeneration. As the number of myonuclei in nuclear chains and in a peripheral location were similar between the genotypes during regeneration, we speculate that myonuclear clustering in regenerating myofibers of ICAM-1-/- mice reflect a compensatory increase in myonuclear accretion. Increased myonuclear accretion in regenerating myofibers of ICAM-1-/- mice we speculate was initiated to alleviate impairments in transcription (present study) and protein synthesis [5].

Regardless of the interpretation of myonuclear clustering during regeneration, the increased number of myonuclei in regenerating myofibers of ICAM-1-/- mice failed to restore transcription (present study), protein synthesis, and myofiber size to wild type levels during regeneration [5]. This failure indicates that ICAM-1 facilitates molecular and cellular processes of regenerating myofiber hypertrophy that are independent or downstream from those that mediate myonuclear accretion during regeneration. Such processes are likely to be complex as ICAM-1 is capable of initiating a diverse array of molecular and cellular processes, as discussed above.

Little is known about cellular and molecular processes that regulate regenerating myofiber maturation after injury. We have begun to advance knowledge in this area by demonstrating a role of ICAM-1 in the regulation of transcription (present study), protein synthesis, and myofiber size during injury-induced muscle regeneration [5]. This knowledge provides a foundation for future investigation into how ICAM-1 facilitates molecular and cellular processes of muscle regeneration. The goal of work in this area is to develop targeted therapeutics for promoting regeneration when aging, trauma, or disease alters intrinsic properties of myogenic cells, as well as the milieu of skeletal muscle.

## Data Availability

Data are available upon request to corresponding author.

## Supplementary Materials

The following supporting information can be downloaded at: https://www.mdpi.com/article/10.3390/ijms23137028/s1.
