# Supplementary figures and images for: Intercellular Adhesion Molecule-1 Enhances Myonuclear Transcription during Injury-Induced Muscle Regeneration
Source: Int J Mol Sci. 2022 Jun 24;23(13):7028. doi: 10.3390/ijms23137028 (PMC9267068; doi:10.3390/ijms23137028)

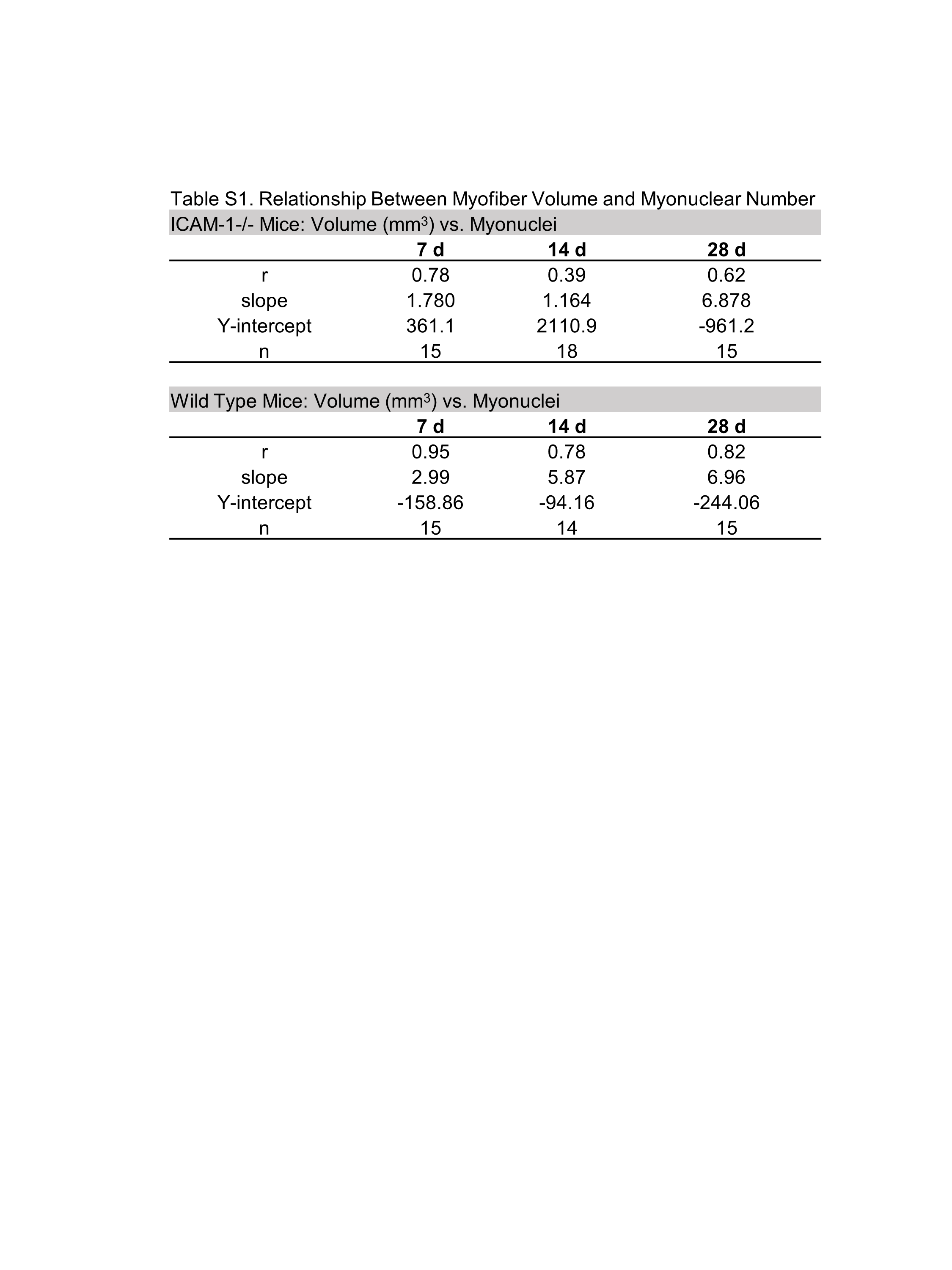

Supplement: Supplementary file 1 [file ijms-23-07028-s001.zip › ICAM-1 Table 1 Nuclei vs Volume.tif]

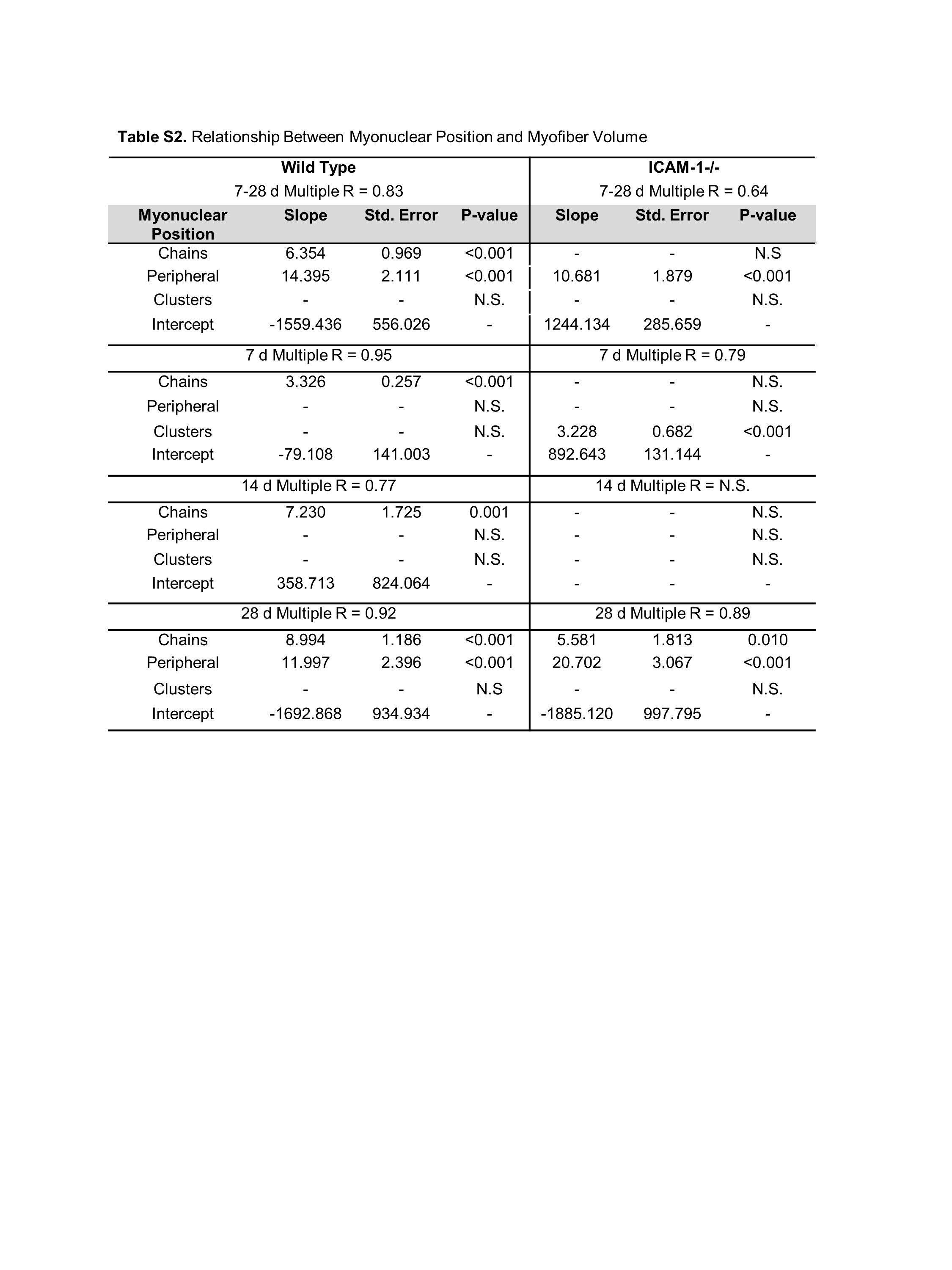

Supplement: Supplementary file 1 [file ijms-23-07028-s001.zip › ICAM-1 Table 2 Multiple Regression - Populations vs Volume.tif]

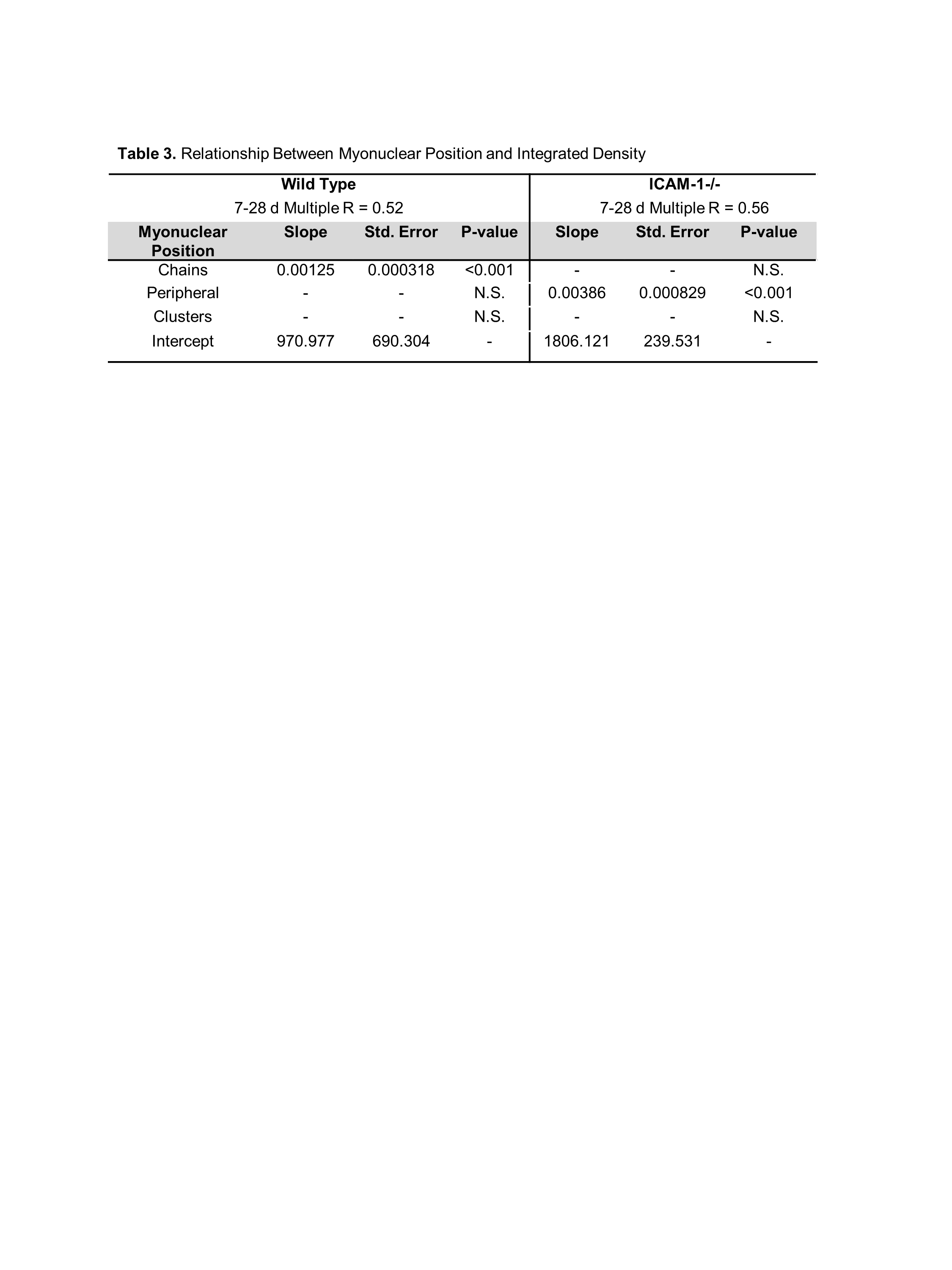

Supplement: Supplementary file 1 [file ijms-23-07028-s001.zip › ICAM-1 Table 3 Multiple Regression - Populations vs ID.tif]
